# Microwave-assisted enhanced activation of date palm leaf char for optimized CO_2_ adsorption

**DOI:** 10.1038/s41598-025-18683-7

**Published:** 2025-10-07

**Authors:** S. K. Al-Janabi, Hind Shabbani, James M. Courtney, Andrew R. Barron, M. R. Othman

**Affiliations:** 1 School of Chemical Engineering, Universiti Sains Malaysia, Engineering Campus, Nibong Tebal, Pulau Pinang, 14300 Malaysia; 2 Municipality of Baghdad, Solid Waste and Environment Office, Baghdad, Iraq; 3 Department of Chemical Engineering, Swansea University Bay Campus, Swansea, SA1 8EN U.K.; 4 College of Engineering, University of AL Qadisiyah, Engineering Campus, AL Diwaniyah, 58002 AL Qadisiyah, Iraq; 5 MiDAS Green Innovations, Swansea, SA1 8RD U.K.

**Keywords:** Activated carbon, Microwave, response surface methodology, CO_2_ capture, Date palm leaves, Environmental sciences, Energy science and technology, Engineering

## Abstract

This study presents a novel hybrid approach for producing mesoporous activated carbon (AC) from date palm leaves by integrating conventional carbonization with microwave-assisted activation. K₂CO₃ was utilized for chemical activation to enhance adsorption performance. Three critical parameters, namely microwave power, residence time, and activation temperature, were investigated focusing on yield, temperature ramp-up time, and CO₂ capture capacity. Results reveal that both microwave power and activation temperature were pivotal in influencing the yield and adsorption efficiency. Microwave-assisted activation (850 W, 500 °C, 7 min) yielded an optimal CO₂ adsorption capacity of 126.7 mg/g at 25 °C and 1 bar, with 78% and 76% reductions in activation energy and processing time, respectively. This method enhanced the textural properties of the activated carbon, increasing the surface area to 411 m²/g and improving pore size distribution, as compared to conventional thermal activation (60 min under CO₂), which achieved only 88 mg/g uptake and a surface area of 286 m²/g. Compared to conventional methods, the hybrid approach reduced energy and time demands to about 25%.

**Supplementary Information:**

The online version contains supplementary material available at 10.1038/s41598-025-18683-7.

## Introduction

Intensified research for sustainable solution that addresses waste management and carbon capture is attributed to escalating global environmental challenges relating to climate change and mounting waste generation. Agriculture waste is considered one of the largest Renewable biomass sources worldwide. In recent years, there has been an increasing focus on environmental sustainability and waste recycling utilizing agricultural byproducts in the Middle East. Date palm waste stands out as a substantial and underutilized resource, generated annually in large quantities. Date palm leaves constitute around 70% of total date palm waste. A comprehensive review of the Scopus database shows that between approximately 860 publications on date palm waste utilization only 20% specifically focus on this abundant resource. This signifies research gap, highlighting the untapped potential of date palm leaves as a sustainable raw material for environmental utilization^1–3^.

The conversion of biomass to activated carbon for water and gas purification is prominent trend to combat environmental challenges. Activated carbon typically involves two main stages: carbonization of the biomass, followed by physical or chemical activation. However, the activation phase faces challenges relating to the requirement of high temperatures over extended periods, resulting in substantial energy consumption^4^. Given the ongoing global energy crisis, there is growing interest in adopting alternative energy sources. Microwave-assisted activation has emerged as a promising alternative for the conventional method.

Microwave heating offers significant advantages over conventional thermal methods by providing rapid, controlled, and uniform heating, offering a cost-effective and environmentally friendly solution for industrial energy demands^5–7^. Microwave-assisted activation generates heat internally through the electromagnetic field interactions with polar molecules via dipolar rotation and molecular vibrations, resulting in uniform heating throughout the material^8^. The internal heating facilitates the controlled movement of volatile components from the hotter interior to cooler regions, minimizing energy losses and promoting the formation of uniform pores. This process reduces the likelihood of undesired secondary reactions^9^. Conventional heating relies on thermal conduction which leads to uneven temperature distribution where the exterior being significantly hotter than the core. As a result, conventional heating is slower and less energy efficient than microwave heating^10^. The main limitations associated with microwaves are attributed to safety hazards requiring proper containment within Faraday cages, material and design limitations that restrict reactor construction, and the risk of equipment damage from improper material selection^11^. Difficulty in controlling heating rate, restrictions relating to the amount of sample utilized, and hot spot formation are reported limitations associated with microwave utilization^12^.

Recent studies have demonstrated the potential of microwave-assisted activation for biomass-derived activated carbon production. Durán-Jiménez et al. (2020) investigated in-situ microwave pyrolysis-activation of pecan nutshells chemically activated with K₂CO₃. Their study demonstrated that the activated carbon achieved a CO₂ capture capacity of 145 mg/g at STP when the pyrolysis temperature reached 375 °C after 6 min of heating at 300 W^13^. However, the self-pyrolysis activation process can sometimes be ineffective, as reported by Biti et al. (2024). Even with a high input power of 1800 W applied to treat microcrystalline cellulose, the temperature failed to exceed 300 °C. This indicates that microwave energy alone may not efficiently carbonize biomass, as different biomass types exhibit varying microwave absorption properties, preventing the achievement of the required pyrolysis and activation temperatures^14^.

**Fig. 1:**
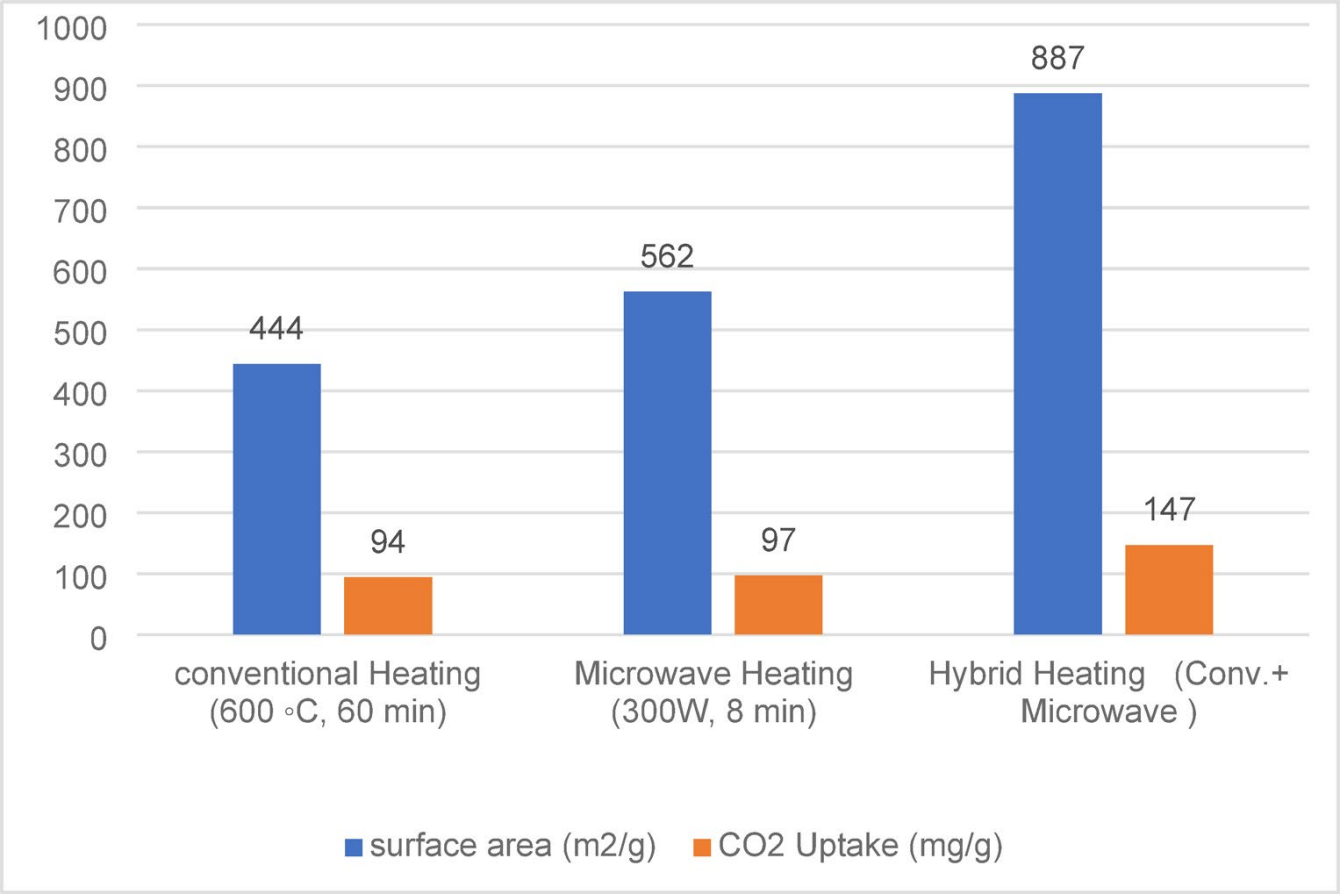
Effect of Heating Mode on Prepared Activated Carbon Surface Area and CO₂ Uptake Adapted from Reference 15. Published under a CC BY license with permission from the copyright holder.

To address this limitation, hybrid heating combining conventional and microwave methods has been proposed to enhance microwave absorption and heat conversion, thereby improving the surface characteristics and adsorption capacity of activated carbon. Figure 1 illustrates the effect of heating mode on surface area and CO₂ uptake, based on data from Durán-Jiménez et al. (2022).AC was prepared by single-step conventional heating, single-step microwave heating, and a two-step hybrid method (conventional + microwave)^15^. This comparison highlights the influence of the heating approach on the textural properties and CO₂ adsorption capacity of the resulting activated carbons, demonstrating that hybrid methods can enhance performance by combining the advantages of both techniques. In particular, the integration of conventional heating with microwave activation during the carbonization step has been proposed as an effective strategy to synergistically exploit the benefits of each method, thereby optimizing energy efficiency and improving the overall quality of the activated carbon^14,16–18^.

Chemical activation plays a pivotal role in enhancing the adsorption capacity of biochar. Potassium carbonate (K₂CO₃) has emerged as a particularly effective activating agent, significantly enhancing both physical and chemical properties of biochar. Abdulhameed et al. (2023) developed mesoporous activated carbon from a mixture of oil palm fronds and palm kernel shells impregnated with K₂CO₃. Using microwave radiation, they achieved a methylene blue removal capacity of 331.6 mg/g^19^. Similarly, Zhu et al. (2018) demonstrated that K₂CO₃ activation significantly improved the physical and chemical properties of biochar, with biochar prepared at 600 °C exhibiting a specific surface area five times greater than that of non-activated biochar. This activated biochar featured an aromatized, non-polar surface conducive to effective adsorption^20^. Wu et al. (2023) studied bamboo shoot shell-derived porous carbons activated with K₂CO₃ for CO₂ adsorption from flue gas. Their findings revealed a substantial increase in specific surface area of approximately 15 times greater than the untreated material with CO₂ adsorption capacity of 151.4 mg/g at 25 °C and 1 bar^21^.

Despite recent literature, there remains a significant gap in research relating to the optimization of activated carbon production from date palm leaves, particularly through the integration of chemical activation with hybrid microwave-assisted thermal treatment. The abundance of date palm waste in Iraq and the broader Middle East region, combined with the demonstrated effectiveness of hybrid activation approaches, presents an opportunity to develop sustainable, high-performance activated carbon materials for CO₂ capture applications.

In this study, this gap is addressed by presenting a novel approach to optimize both the quantity and quality of activated carbon derived from date palm leaves, an abundant yet underutilized biomass resource in Iraq. By combining chemical activation with K₂CO₃ and thermal activation through microwave-assisted techniques, we aim to improve the pore size distribution and carbon structure, thereby enhancing CO₂ adsorption capacity. Using the Box-Behnken design, the study evaluated the effects of three critical parameters, namely microwave power, residence time, and activation temperature, on yield, temperature ramp-up time, and CO₂ capture capacity. A matrix of 13 experimental runs was generated, and the results were then analyzed via analysis of variance (ANOVA). The process would then be optimized by optimizing independent factors. This study not only provides a solution for date palm waste management but also contributes to the development of cost-effective, sustainable carbon capture technologies essential for addressing climate change challenges.

## Materials and methods

### Precursors

In this study, date palm leaf waste collected from farmland in Baghdad, Iraq, was pretreated for subsequent use. The leaves were first chopped into 1–3 cm pieces and rinsed with distilled water to remove the initial surface impurities and dirt. Afterward, the leaves were dried in an oven at 110 °C overnight. Once dried, the leaves were ground and sieved to achieve a particle size range of 0.2–0.5 mm and then stored in a sealed container to maintain their condition. Potassium carbonate (K₂CO₃), purchased from Sigma-Aldrich, Spain, was used for the activation process.

### Bio-char Preparation and activation

**Fig. 2:**
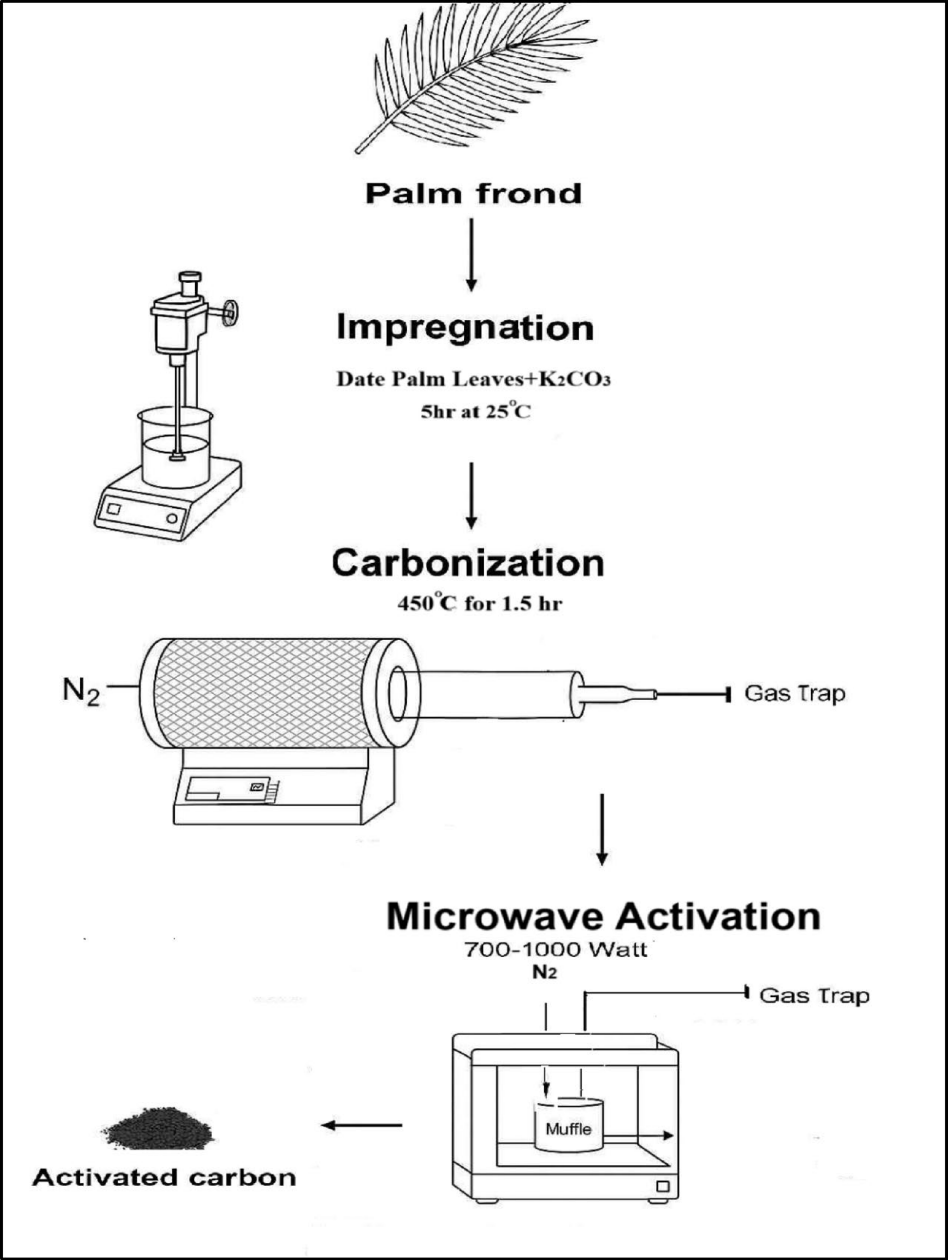
Schematic diagram of the activated carbon preparation process.

Activated carbon (AC) samples were prepared through a three-step process: potassium carbonate impregnation, carbonization, and thermal activation using microwave. The entire process is schematically illustrated in Fig. 2. Initially, 2 g of pre-treated date palm leaves (DPL) were impregnated with a 50 g/L K₂CO₃ solution at a weight ratio of 1.5:1 (K₂CO₃: DPL) for 5 h at room temperature. Following impregnation, the mixture was filtered and dried at 105 °C for approximately 16 h. Following drying, the samples were placed in an alumina crucible at the center of a glass reactor (100 cm length and 5.5 cm diameter) inside a tube furnace for carbonization. The carbonization process was carried out at 450 °C for 1.5 h under a nitrogen flow of 200 mL/min. Although the precursor was impregnated with K₂CO₃, the thermal treatment at 450 °C is below the effective activation range (500–700 °C). Thus, this step is considered carbonization with minimal activation^22^.

**Fig. 3:**
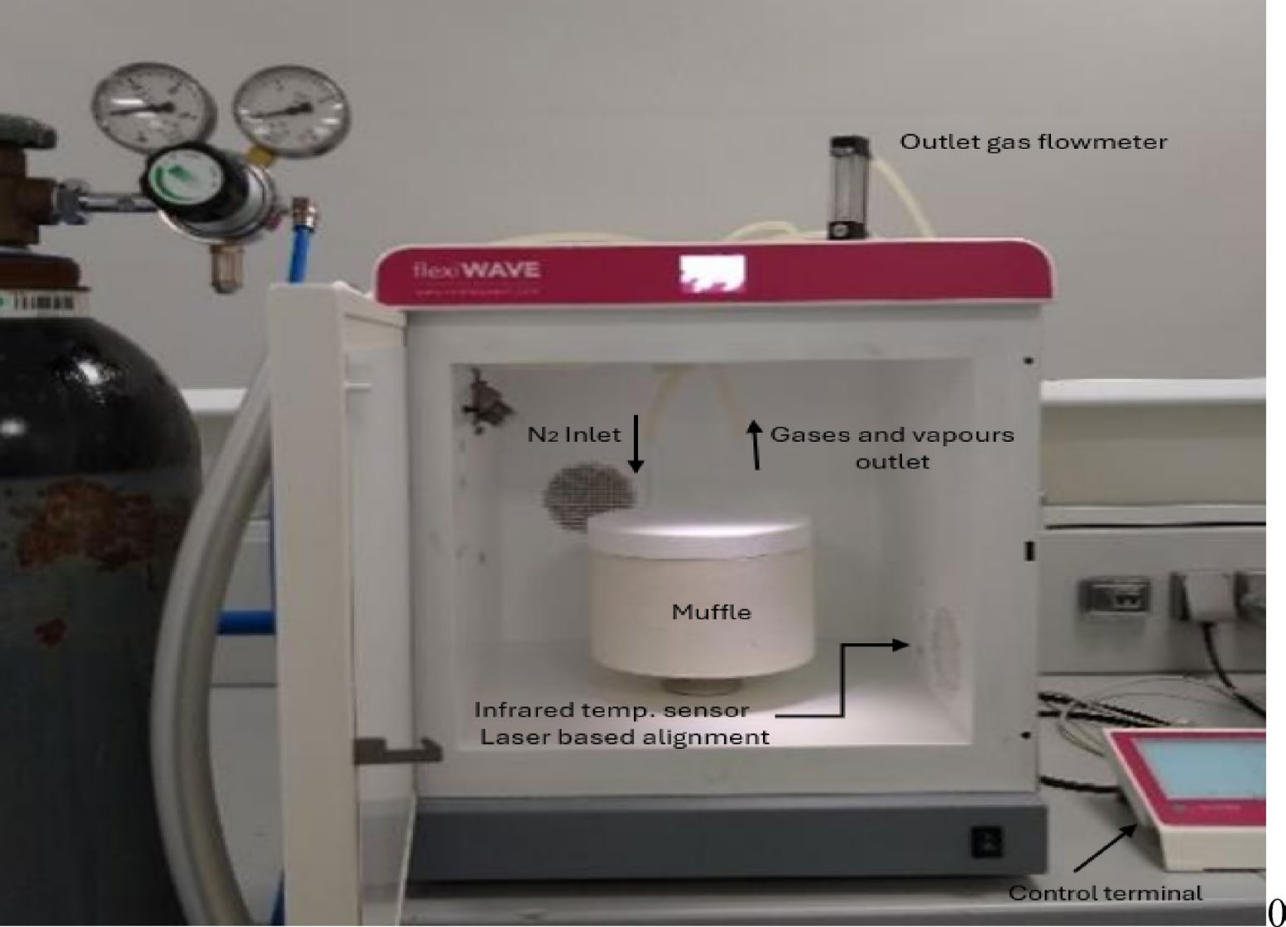
PYRO Advanced Microwave Muffle Furnace and its Components Used for DPL Char Activation, Supplied by Milestone Company.

After carbonization, the samples were allowed to cool in a nitrogen atmosphere. The carbonized samples were then subjected to the final activation stage using a Milestone PYRO advanced microwave muffle furnace Fig. 3 at varying temperatures, power levels, and residence times. The temperature during microwave-assisted activation was controlled using an infrared (IR) temperature sensor with laser-based alignment, integrated into the microwave system, as shown in Fig. 2. This non-contact IR sensor allows for real-time monitoring and accurate control of the sample temperature throughout the activation process, ensuring reproducibility and safety during thermal treatment. Microwave has presented itself as an alternative heating method with efficient energy pyrolysis. By preventing unwanted secondary reactions, microwave volumetric heating ensures consistent activation conditions throughout the precursor material, resulting in more uniform pore development, better surface area characteristics, and superior quality activated carbon with a reduced risk of surface overheating or incomplete interior activation^23^.

The resulting product was washed multiple times with distilled water until a neutral pH of 7 was reached, effectively removing any residual ash or unreacted chemicals. The activated carbon was prepared in two separate batches using identical procedures to ensure uniformity.

The total yield was calculated using Eq. 1,

1 $$\text{Yield}\:{\%}=(\text{Wf/W0})\times100$$

Where Wf is the final weight and W0 is the initial weight of the sample.

The addition of K₂CO₃ will initiate a redox reaction with carbonaceous materials, where it is oxidized into CO, yielding porosity in the remaining carbon, as shown in Eq. 2^20,24^. The removal of tarry materials leads to the development of meso- and micropores at a temperature below 600 °C^20^. The produced CO also contributes to the gasification process. The expansion and disruption of the carbon matrix will be caused by elemental K intercalating within it.

2 $$\text{K}_2\text{CO}_3+2\:\text{C}\rightarrow2\text{K}+3\:\text{CO}$$

### Materials characterization

Scanning Electron Microscopy coupled with Energy Dispersive X-ray Spectroscopy (SEM-EDX) was performed using a HITACHI TM3030 Plus to examine the surface morphology, conduct elemental mapping, and provide quantitative analysis of the sample composition. A NOVA 2000e Surface Area and Pore Size Analyzer was used to assess the textural properties of the prepared date palm leaf activated carbon (DPL-AC) through nitrogen adsorption at −196.15 °C, following sample degassing at 110 °C for 12 h. The specific surface area and pore distribution were determined using the Brunauer, Emmett, and Teller (BET) and Density Functional Theory (DFT) methods, respectively. The total pore volume was calculated at a relative pressure of 0.98^25^. Thermogravimetric analysis (TGA) was performed under both argon and carbon dioxide atmospheres to evaluate CO₂ uptake capacity. Samples were placed in an alumina crucible and heated to 105 °C under an argon atmosphere to remove moisture. After the moisture evacuation step, the samples were cooled to 30 °C under atmospheric pressure. Once at 25 °C, the gas flow was switched to CO₂, and the samples were exposed to CO₂ for one hour to assess their adsorption behaviour. The CO₂ uptake was calculated based on the weight difference such as shown by Eq. 3, where *X*_*evacuated*_ is the weight after purging (mg) and *X*_*final*_ is the weight after CO₂ adsorption (mg).

3 $$\:C{O}_{2}uptake\:=\frac{{{X}_{final}-X}_{evacuated}}{{X}_{evacuated}}$$

For future research, X-ray Photoelectron Spectroscopy (XPS) and X-ray Diffraction (XRD) analyses are recommended to enhance the comprehensive understanding of the work^26^.

### Experimental design of response surface methodology (RSM)

Design-Expert 13, a specialized software for optimizing and formulating experimental design, was used. The Box-Behnken design was selected in this study with 3 factors and 3 responses as illustrated in Table 1. This design enables effective process optimization through second-order quadratic modelling, as delineated in Eq. 4^19^.

4 $$Y=\beta_0+\sum\beta_iX_i+\sum\beta_{ii}X_i^2+\sum\sum\sum\beta_{ij}X_iX_j$$

where *Y* represents the response; *X*_*i*_ and *X*_*j*_ are the independent factors; *β*_*0*_ denotes the intercept; *β*_*i​*_ indicates the linear effect; *β*_*ii​*_ represents the interaction effect between the independent factors; and *β*_*ij​*_ signifies the quadratic effect.

A matrix of 14 experimental runs is generated by Design-Expert 13 to analyse the results using analysis of variance (ANOVA). This process determines the most appropriate fitting model and derives equations that precisely represent yield, CO₂ removal, and the ramp time required for the char to reach the desired activation temperature. Subsequently, the independent factors are optimized to enhance responses, and the validity of the fitted model is rigorously evaluated to ensure its reliability in predicting outcomes. RSM analyses were performed using Design Expert version 13 (Stat-Ease Inc., USA). The software was accessed under Universiti Sains Malaysia’s institutional license. Software details: https://www.statease.com/software/design-expert/.

**Table 1:** Independent factors, their levels, and the responses studied using the Box-Behnken design.

Independent factors	Unit	Level (−1)	Level (0)	Level (+ 1)	Dependent factors	Unit
Microwave power	Watt	700	850	1000	Yield	%
Residence time	min	4	7	10	CO_2_ Uptake	mg/g
Activated Temp.	^○^C	400	500	600	Ramp time	min

## Results and discussion

### Box-Behnken model analysis

The validity of the response results obtained from the Box–Behnken model was confirmed through ANOVA and lack-of-fit analysis. The corresponding experimental design and data are detailed in Table 2, which presents the tabulated design matrix suggested by Design Expert 13.

The statistical analysis presented in Table 3 demonstrates the model’s effectiveness in predicting CO₂ uptake, as described by the quadratic Eq. 5. The model shows a high coefficient of determination (R² = 0.9174), with good agreement between the adjusted R² (0.8658) and the predicted R² (0.7344), indicating strong explanatory and predictive capability. An F-value of 17.78 and a p-value of 0.0004 further confirm the model’s statistical significance.

Additional indicators support the model’s adequacy, including a low standard deviation (5.36), a mean response of 112.93, a coefficient of variation (CV) of 4.74%, and an Adequate Precision value of 13.0277 well above the recommended threshold of 4. The CV being below 5% reflects excellent precision and reproducibility. Although the Prediction Error Sum of Squares (PRESS) was 738.05, this value remains acceptable given the response scale and the model’s overall performance, indicating strong predictive reliability.

5 $$\text{CO}_2\text{Uptake}=126.7-13.875\text{A}-0.25\text{B}-4.63\text{C}-11.05\text{A}^2-13.05\text{C}^2$$

According to the F-values presented in Table 3, microwave power was identified as the most influential factor in enhancing CO_2_ adsorption capacity, followed by activation temperature, while activation time had a comparatively minor effect. These results confirm the reliability and robustness of the quadratic model in capturing CO_2_ adsorption behavior under the tested conditions.

**Table 2:** Tabulated design matrix suggested by design expert 13.

Run	Microwave power (Watt)	Residence time (min)	Activation Temperature (°C)	Yield (%)	CO_2_ Uptake (mg/g)	Ramp time (min)
1	850	4	400	16	28	123
2	1000	10	500	18	23	107
3	850	4	600	19	37	103
4	850	7	500	20	32	129
5	1000	4	500	20	24	98
6	850	10	600	19	38	108
7	850	7	500	17	36	130
8	700	4	500	15	42	130
9	1000	7	600	20	27	86
10	700	10	500	18.7	38	122
11	850	10	400	17	27	115
12	700	7	400	14	35	123
13	700	7	600	18	48	117
14	1000	7	400	21	20	90

**Table 3:** ANOVA analysis for the reduced quadratic model of the CO_2_ uptake response.

Source	Sum of squares	Degree of freedom	Mean square	F- value	*P*-value
Model	2549.48	5	509.90	17.78	0.0004
A-MW Power	1540.13	1	1540.13	53.70	< 0.0001
B-Resident Time	0.5000	1	0.5000	0.0174	0.8982
C-Activation Temp.	171.13	1	171.13	5.97	0.0404
A^2^	407.01	1	407.01	14.19	0.0055
C^2^	567.68	1	567.68	19.79	0.0021
Residual	229.45	8	28.68		
Lack of Fit	228.95	7	32.71	65.41	0.0949
Pure Error	0.5000	1	0.5000		
Cor Total	2778.93	13			

Table 4 confirms that the two-factor interaction (2FI) model offers the best fit to the experimental yield response data compared to other models. This conclusion is supported by the model fit summary, which reports a statistically significant sequential p-value (0.0638), a high lack-of-fit p-value (0.9537), and close agreement between the adjusted R^2^ and predicted R^2^ values indicating strong consistency between the model’s explanatory and predictive capabilities.

**Table 4:** Summary of model fit statistics for the yield response.

Source	Sequential *p*-value	Lake of fit *p*-value	Adjusted *R* ^2^	Predicted *R* ^2^	
Linear	0.0320	0.8224	0.4406	0.1567	
2FI	**0.0638**	**0.9537**	**0.6995**	**0.5051**	**Suggested**
Quadratic	0.9125	0.8628	0.5332	−0.3068	
Cubic	0.8628		0.0743		Aliased

Additional statistical metrics further validate the adequacy of the reduced 2FI model. The model achieved an R^2^ value of 0.8336, indicating that approximately 83.36% of the variability in yield is explained by the model. The adjusted R^2^ (0.7296) and predicted R^2^ (0.5336) values show reasonable agreement, suggesting good model reliability without overfitting. A standard deviation of 1.06, a mean response of 18.05, and a coefficient of variation (CV) of 5.9% all point to acceptable variability and precision.

Moreover, the Adequate Precision value of 9.5633 exceeds the minimum recommended threshold of 4, demonstrating a strong signal-to-noise ratio. The model’s Prediction Error Sum of Squares (PRESS) was 25.4, further supporting its predictive capability. Based on these indicators, the reduced 2FI model was selected as the most appropriate representation of the system behaviour. The corresponding coded equation is presented in Eq. 6.

6 $$\text{Yield}=18.05+1.663\:\text{A}+0.338\text{B}+1\:\text{C}-1.425\text{AB}-1.25\text{AC}$$

As indicated by the ANOVA results (Table 5), microwave power had the most significant effect on yield, with an F-value of 19.52 and a p-value of 0.0022, confirming its strong influence. Activation temperature showed a moderate effect (F = 7.064), with a p-value of 0.0289, indicating statistical significance at the 5% level, though its influence was less pronounced. In contrast, residence time was not statistically significant, with an F-value of 0.805 and a p-value of 0.396, indicating a negligible effect on yield.

Importantly, interaction effects were statistically significant. The interaction between microwave power and activation temperature (AC) had a p-value of 0.0467, while the interaction between microwave power and residence time (AB) had a p-value of 0.028. These results underscore the importance of including interaction terms in the predictive model to accurately represent the system’s behaviour.

**Table 5:** ANOVA analysis for reduced 2FI model of the activated carbon yield response.

Source	Sum of squares	Degree of freedom	Mean square	F- value	*P*-value
Model	45.39	5	9.08	8.02	0.0056
A-MW Power	22.11	1	22.11	19.52	0.0022
B-Resident Time	0.9112	1	0.9112	0.8046	0.3959
C-Activation Temp.	8.00	1	8.00	7.06	0.0289
AB	8.12	1	8.12	7.17	0.0280
AC	6.25	1	6.25	5.52	0.0467
Residual	9.06	8	1.13		
Lack of Fit	4.56	7	0.6514	0.1448	0.9660
Pure Error	4.50	1	4.50		
Cor Total	54.45	13			

Furthermore, ramp-up time, the duration required for the material to absorb microwave energy and reach the desired activation temperature, was analysed as a critical indicator of microwave-assisted activation efficiency. The data exhibited a strong fit to the linear model (Eq. 7), with a high coefficient of determination (R² = 0.9694), and close agreement between the adjusted R² (0.9602) and predicted R² (0.9419), confirming the model’s robustness. Additional statistical parameters, including a low standard deviation (1.59), a low coefficient of variation (CV = 4.89%), a high Adequate Precision value (32.1281), and a PRESS value of 47.80, further validate the model’s adequacy. As summarized in Table 6, ANOVA analysis revealed that microwave power had the most significant effect on ramp-up time (F = 236.36, *p* < 0.0001), followed by activation temperature (F = 79.43, *p* < 0.0001). Conversely, residence time had no statistically significant influence (F = 1.24, *p* = 0.2913). These results highlight the dominant roles of microwave energy and activation temperature in enhancing heating efficiency and process control, which are essential for optimizing the energy demand and economics of activated carbon production^27^.

7 $$\text{Ramp}\:\text{time}=32.43-8.625\:\text{A}-0.62\text{B}+5\:\text{C}$$

**Table 6:** ANOVA analysis of factors affecting Ramp-Up time during microwave activation.

Source	Sum of squares	Degree of freedom	Mean square	F- value	*P*-value
Model	798.25	3	266.08	105.68	< 0.0001
A-MW Power	595.13	1	595.13	236.36	< 0.0001
B-Resident Time	3.13	1	3.13	1.24	0.2913
C-Activation Temp.	200.00	1	200.00	79.43	< 0.0001
Residual	25.18	10	2.52		
Lack of Fit	20.68	9	2.30	0.5106	0.8048
Pure Error	4.50	1	4.50		
Cor Total	823.43	13			

### Model interactions and optimum conditions validation

**Fig. 4:**
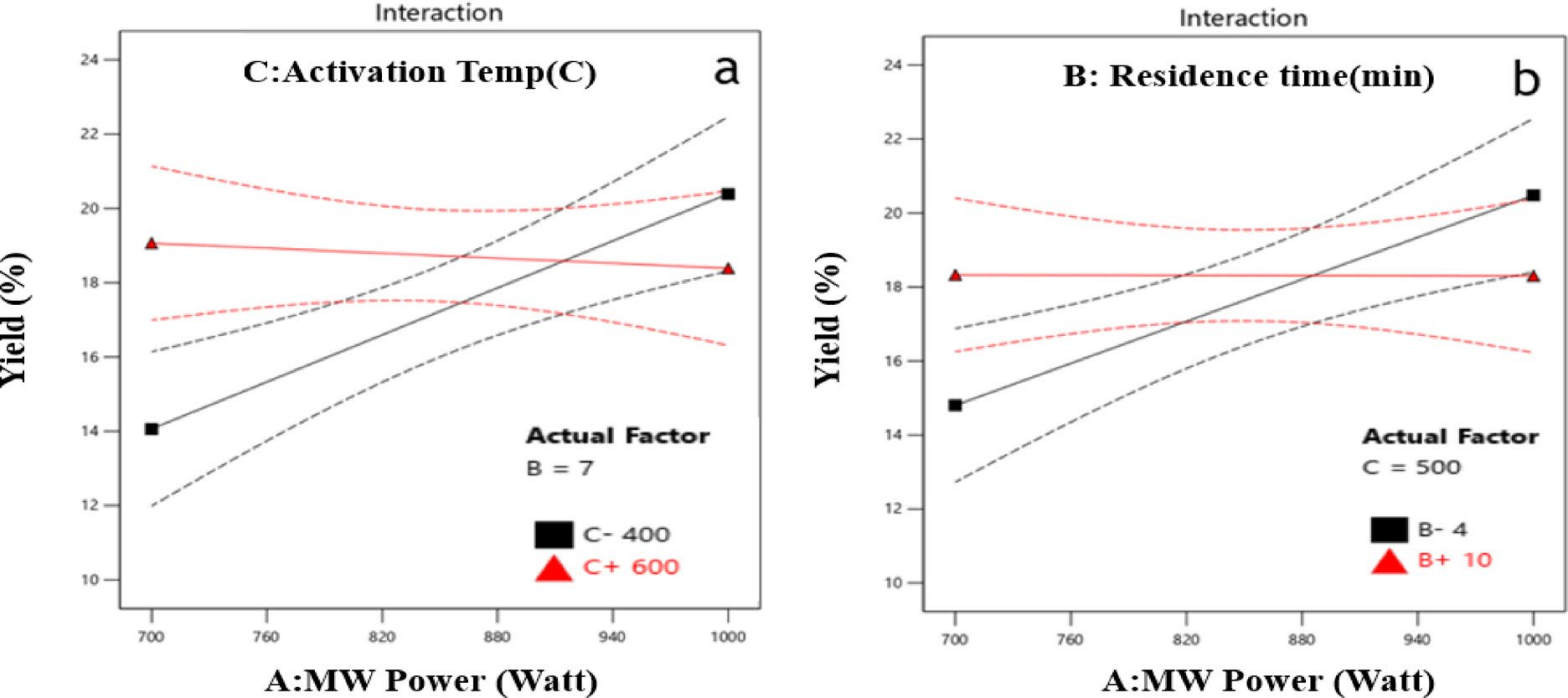
Interactions between factors and their effect on AC Yield (**a**) activation temperature-MW power interaction. (**b**) Residence time-MW power interaction.

As indicated by the ANOVA analysis, microwave power has the most significant impact on both overall yield and CO₂ adsorption capacity, followed by activation temperature. The effect of residence time is minimal, with only limited interactions with microwave power affecting the yield of the prepared AC. Focusing on the AC yield at a constant residence time, an inverse relationship between microwave power and activation temperature is observed. At lower temperatures, the activation process is less intense; however, increasing the microwave power compensates for the lower thermal energy, facilitating the removal of residual volatile materials and promoting the reaction between carbon and K₂CO₃. This enhances both porosity and overall yield. In contrast, continuous increases in energy at higher activation temperatures negatively impact the yield by accelerating the reaction rate, which can result in excessive carbonization and the burning of material as demonstrated in Fig. 4^17^.

At a moderate activation temperature of 500 °C and a short residence time, increasing microwave power for a brief period provides sufficient energy to accelerate the activation process, thereby enhancing pore development and facilitating volatile release (see Fig. 4b). However, further prolonged residence times has a neglected effect, as the reactions are largely complete, and the material has already reached its maximum activation potential.

**Fig. 5:**
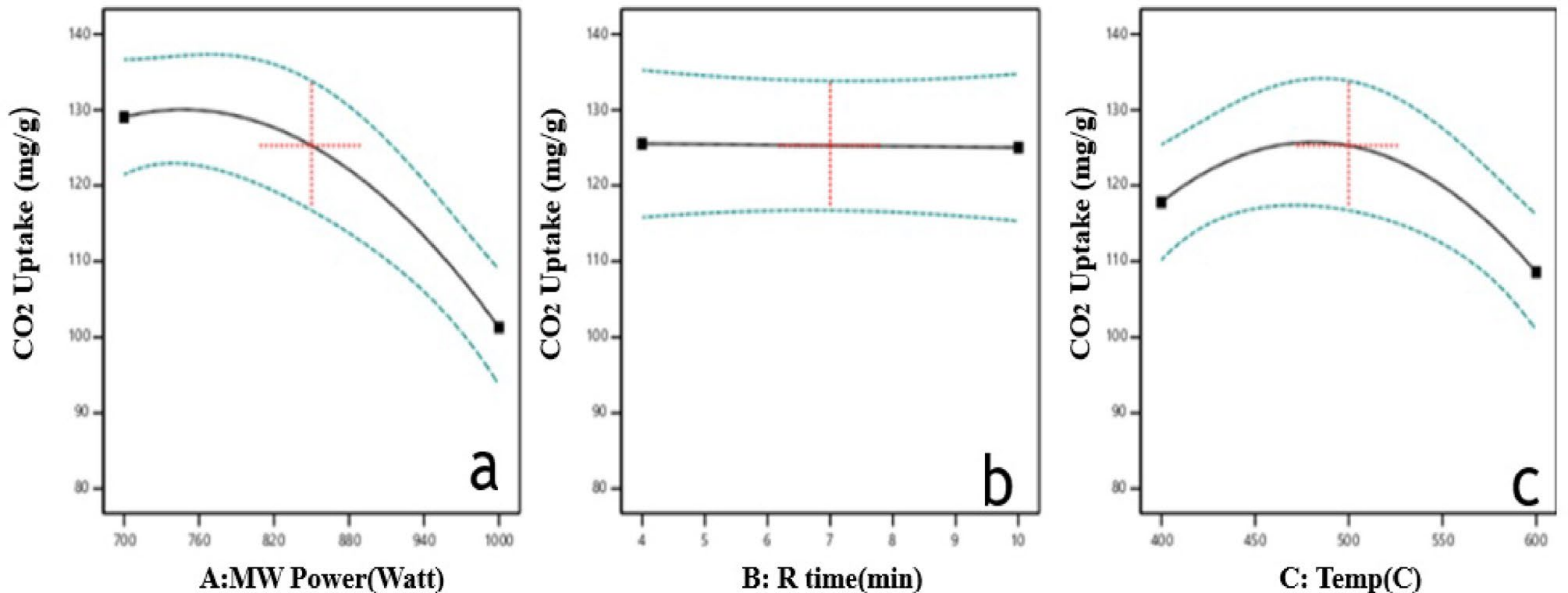
ANOVA Analysis of factors affecting CO₂ uptake (**a**) activation temperature, (**b**) microwave power, and (**c**) residence time.

According to the RSM model optimization analysis, the optimal conditions for microwave-assisted activation were 850 W, 500 °C, and 7 min, as illustrated in Fig. 5.

Microwave-assisted activation enables lower activation temperatures (e.g., 500 °C compared to 600 °C in conventional heating) by providing rapid, uniform, and volumetric heating. This efficient energy transfer reduces thermal losses and produces localized hotspots that accelerate the activation process, thereby enhancing the interaction between the activating agent (K₂CO₃) and the carbon structure^28^.

As shown in Fig. 5a, The selection of 850 W as the optimal power was based on a multi-objective optimization using Response Surface Methodology (RSM), which considered not only the CO₂ uptake but also other critical factors such as carbon yield and process ramp-up time. While a slightly lower power might maximize adsorption, 850 W represents the best overall compromise among these competing objectives for practical application^8,9,29^.

Similarly, Fig. 5b indicates that increasing residence time beyond 7 min yields minimal improvement in CO₂ adsorption. This suggests that most activation reactions occur rapidly under microwave conditions, and prolonged exposure may not significantly benefit the outcome and could even contribute to structural degradation.

Figure 5c demonstrates that CO₂ uptake increases with temperature up to 500 °C, indicating improved pore formation. However, at 600 °C, a noticeable drop is observed, likely due to pore widening, collapse, or burn-off, which reduces the material’s microporosity and effectiveness for CO₂ capture.

Therefore, optimizing microwave power, temperature, and time is critical to achieving high AC yield, maximizing CO₂ adsorption capacity, and ensuring energy-efficient processing.

### Characterization of date palm leaves activated carbon

**Fig. 6:**
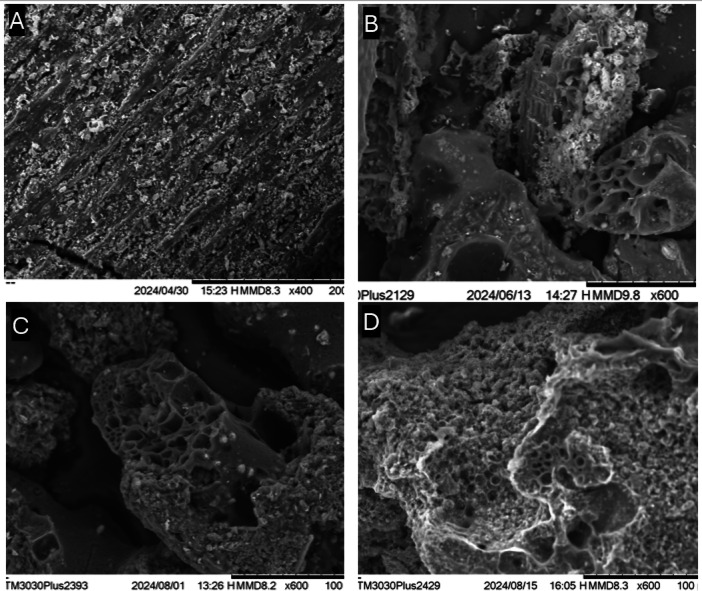
Scanning electron microscopy (SEM) images of date palm leaf-derived samples at various processing stages: (**A**) Raw date palm leaves (DPL); (**B**) K₂CO₃-impregnated feedstock after carbonization (5 °C/min ramp rate, 450 °C for 1.5 h under N₂); (**C**) Activated carbon produced via conventional heating (K₂CO₃, 5 °C/min, 600 °C for 60 min under N₂); (**D**) Activated carbon produced under optimal microwave-assisted conditions (K₂CO₃, 850 W, 500 °C for 7 min under N₂).

The scanning electron microscopy (SEM) images of date palm leaves (DPL) and date palm leaves activated carbon (DPL-AC) are presented in Fig. 6.

Figure 6A reveals that raw date palm leaves (DPL) exhibit a smooth, compact surface morphology, typical of untreated lignocellulosic biomass with minimal inherent porosity. After pre-impregnation with K₂CO₃ and carbonization at 450 °C for 1.5 h under a nitrogen atmosphere, Fig. 6B displays significant morphological transformation. The surface appears more fractured and begins to exhibit pore-like features, indicating partial decomposition of hemicellulose and cellulose. This structural evolution is driven by the catalytic role of K₂CO₃, which facilitates the removal of volatile matter and promotes early-stage pore development by enhancing the decomposition kinetics and rearrangement of carbon structures, even before full activation occurs^30^.

Upon full activation, the morphological differences are further amplified. Figure 6C presents the surface of activated carbon produced via conventional thermal activation (K₂CO₃, 5 °C/min, 600 °C for 60 min under N₂), showing modest pore formation and surface roughness. In contrast, Figure 6D illustrates the substantial improvement in porosity achieved through microwave-assisted activation (K₂CO₃, 850 W, 500 °C for 7 min under N₂). The rapid and volumetric heating provided by microwave irradiation enhances devolatilization and pore propagation, yielding a more interconnected, mesoporous network with higher surface area and improved CO₂ adsorption capacity. These observations confirm the synergistic effect of chemical activation with K₂CO₃ and microwave-assisted heating in tailoring the porosity and textural properties of bio-derived activated carbon, even under relatively mild processing conditions.

In contrast, conventional heating was applied to the same K₂CO₃-impregnated sample at 600 °C for 1 h, with a heating rate of 5 °C/min under a CO₂ atmosphere resulted in fewer and larger pores, demonstrating the superior efficiency of microwave activation in enhancing pore structure.

The energy-dispersive X-ray spectroscopy (EDX) analysis of the modified surface, as shown in Table 7, reveals significant changes in elemental composition, reflecting substantial surface modifications. Specifically, there is a reduction in carbon content, which can be attributed to the release of gases such as CO₂, CO, and CH_4_ during the activation process. Concurrently, the concentrations of silicon, aluminium, and calcium increase, as these elements are released at higher temperatures. The addition of K₂CO₃ increases both oxygen and potassium content. During activation, potassium reacts at lower temperatures to form water-soluble compounds that are largely removed during washing. However, a significant amount of potassium remains concentrated on the surface due to its strong interaction with the carbon matrix during the activation process^28^.

**Table 7:** Comparison of EDX elemental composition of DPL before and after modification.

Element	Weight%	
	DPL	DPL-AC
C	78.1	62.33
O	18.2	21.12
Al	0.5	0.7
Si	1.7	5.07
K	0.3	6.16
Ca	0.5	4.36
Mg	0.3	0.26
Cl	0.4	-
Total	100.00	100.00

**Fig. 7:**
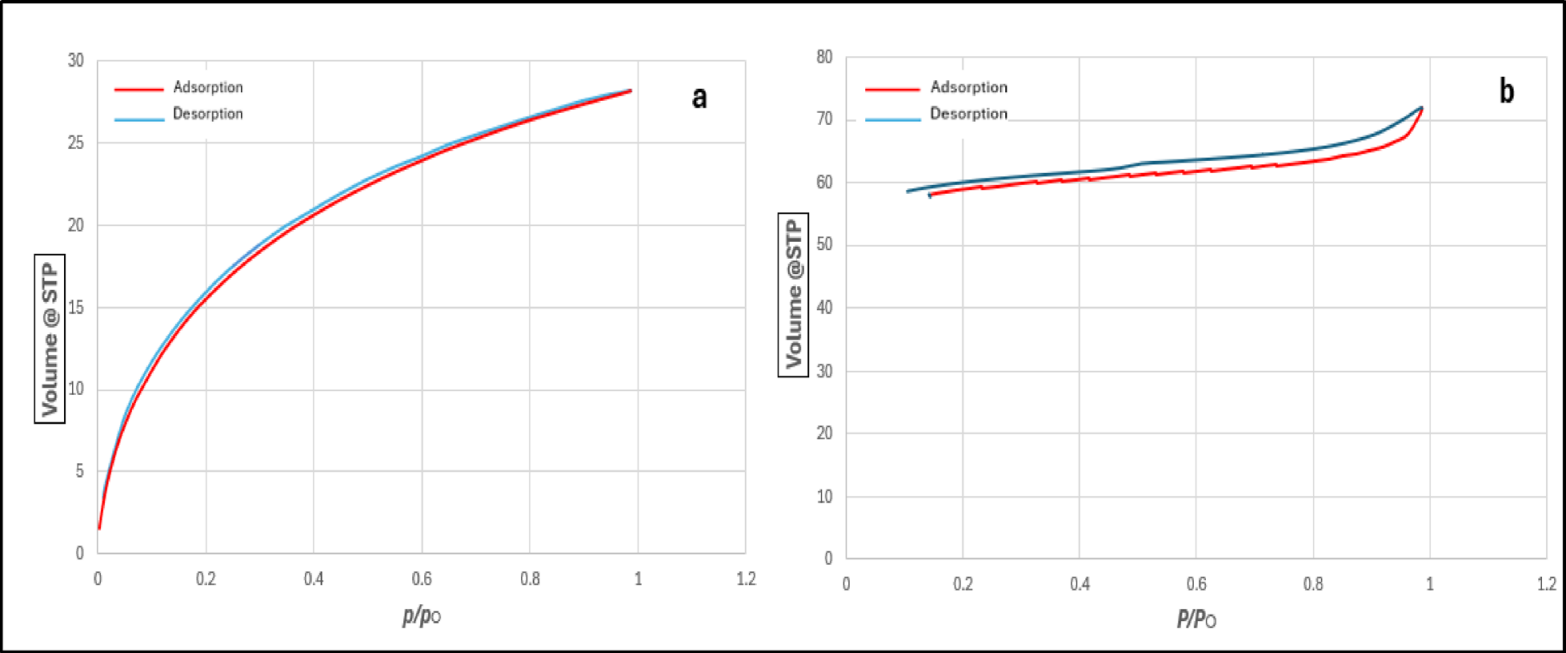
Adsorption–Desorption Isotherms of DPL-Derived Activated Carbon Prepared under Optimum Operational Conditions: (**a**) CO₂ Adsorption at 0 °C and (**b**) N₂ Adsorption at − 196 °C.

As shown in Fig. 7, the CO₂ adsorption isotherm at 0 °C (Fig. 7a) exhibits Type I behaviour, which is indicative of microporous structures and confirms the material’s suitability for CO₂ capture due to strong adsorbate–adsorbent interactions in narrow pores^31^. In contrast, the N₂ adsorption isotherm at 77 K (Fig. 7b) reveals a Type IV profile with a pronounced hysteresis loop, typical of mesoporous materials with pore sizes in the range of 20–500 Å^32^. This distinction arises from the different diffusion behaviours of CO₂ and N₂ at their respective analysis temperatures.

**Fig. 8:**
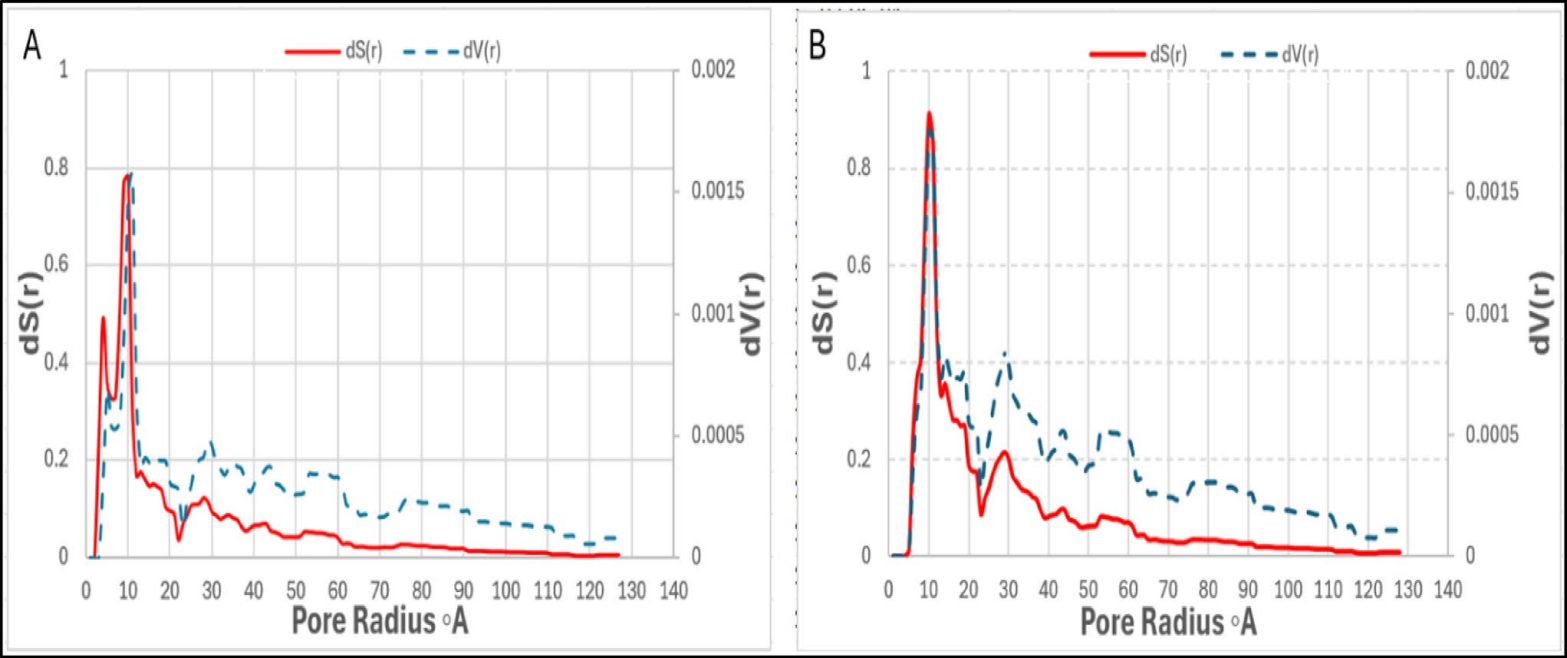
Pore size distribution of DPL-based activated carbon prepared under optimum conditions, as determined by (**A**) N₂ adsorption at 77 K and (**B**) CO₂ adsorption at 0 °C.

These observations are further supported by the pore size distribution profiles presented in Fig. 8. The N₂-derived distribution (Fig. 8A) shows a broad contribution from mesopores ranging from approximately 20 to over 100 Å, confirming the presence of a mesoporous network. In contrast, the CO₂-derived distribution (Fig. 8B) reveals a dominant peak below 10 Å, indicating the prevalence of narrow micropores. Together, these results indicate a hierarchical porous structure comprising both micro- and mesopores. Such dual porosity enhances the performance of the activated carbon, where micropores dominate CO₂ adsorption and mesopores facilitate gas transport and accessibility^33^.

Table 8 presents a comparative analysis of the textural properties of activated carbon produced via conventional heating and hybrid microwave-assisted activation. BET and DFT analyses were used to assess specific surface area, pore volume, and pore size distribution across a pressure range of 110 to 760 Torr, highlighting the enhanced porosity achieved through microwave-assisted activation.

The synergistic combination of K₂CO₃ activation and microwave-assisted heating led to a marked enhancement in both the surface area and CO₂ adsorption capacity of the resulting activated carbon. The raw date palm leaves initially exhibited a low BET surface area of approximately 4 m²/g. Upon 1.5 h of carbonization at 450 °C, this value increased to 35 m²/g, accompanied by a CO₂ uptake of roughly 20 mg/g, reflecting the early-stage impact of thermal decomposition and the catalytic influence of K₂CO₃ pre-impregnation. Subsequent conventional activation under CO₂ flow at 600 °C for one hour further improved the material’s characteristics, yielding a BET surface area of 286 m²/g and a CO₂ adsorption capacity of 88 mg/g. In contrast, microwave-assisted activation under optimized conditions (850 W, 500 °C, 7 min) resulted in significantly enhanced porosity and surface accessibility, achieving a surface area of 411 m²/g and a CO₂ uptake of 126.7 mg/g. This improvement is primarily attributed to the rapid, volumetric heating nature of microwave energy, which facilitates efficient mesopore development, enhances diffusion pathways, and increases the availability of active adsorption sites.

**Table 8:** Comparative summary of activation conditions and outcomes for conventional heating and microwave-assisted (Hybrid) methods.

Specification	Unit	Conventional heating	Microwave heating
Optimum Temp.	^○^C	600	500
Activation time	min	60	7
BET Surface Area	m^2^/g	286	411
Pore size distribution	-	Macro-Meso	Meso-Micro
Yield	%	18.8	18.05
CO_2_ uptake	mg/g	88	126.7

Heating methodology is widely recognized as a key factor governing pore structure evolution in activated carbon, irrespective of the biomass precursor or activating agent employed. Conventional heating typically involves extended ramp-up periods to attain the desired activation temperature, allowing for controlled devolatilization and pore formation. Conversely, the rapid and often uncontrolled heating rate associated with microwave-assisted carbonization can lead to premature volatilization, potentially degrading structural integrity and limiting catalyst efficiency. This observation aligns with findings reported by Zaini and Kamaruddin (2013) and Baytar et al. (2018)^33,34^, who noted similar trade-offs in carbon quality due to abrupt thermal gradients during microwave treatment.

Microwave heating during carbonization presents challenges due to the limited microwave absorption capacity of biomass. To address this, a catalyst was used to enhance absorption, providing indirect heating to the biomass via conduction and convection. Date palm leaves, when carbonized under moderate operational conditions, performed better than other biomass precursors. In contrast, while Elega biomass required a lower carbonization temperature, it still demanded extensive pretreatment and did not yield optimal results. Therefore, date palm leaves were an excellent candidate for activated carbon production. They require minimal pretreatment and moderate conditions, and, importantly, are abundant in large quantities, especially in the Middle East, making them a sustainable and cost-effective source for AC preparation.

By integrating conventional carbonization with microwave activation, the benefits of both methods are leveraged. This approach enhances the char’s microwave absorption, followed by rapid activation in a microwave furnace for a short duration. Durán (2022) demonstrated that Pecan nutshell conventional heating (10 °C/min to 600 °C for 60 min, followed by 60 min activation) resulted in a CO₂ uptake of 94 mg/g at 25 °C and 1 bar. Single-stage microwave pyrolysis showed a slight improvement, with 97 mg/g CO₂ uptake. However, the hybrid system significantly increased CO₂ adsorption to 146 mg/g^15^. In our study, conventional carbonization (450 °C, 10 °C/min, 90 min) and 60 min under CO₂ activation yielded 88 mg/g CO₂ uptake at 1 atm and 25 °C, while combining conventional carbonization with microwave activation (850 W, 7 min, and 500 °C) increased CO₂ uptake to 126.7 mg/g.

The pyrolysis conditions are influenced by the biomass source, with optimal parameters determined through Thermogravimetric Analysis (TGA) of the raw material, as outlined in our previous work. Despite biomass variations, the microwave activation process follows a similar pattern because the primary structure of the carbon is formed during carbonization. The optimal conditions for microwave activation depend more on the type of activating agent, the microwave power needed to reach the desired temperature, and the reaction time required for complete activation, rather than the biomass source itself. These factors govern the interaction between the activating agent and carbon, ultimately affecting the adsorptive properties and structural characteristics of the activated carbon.

Table 9 presents a comparative analysis of different raw biomass sources, their corresponding optimal carbonization conditions, and the influence of microwave power and holding time during the microwave-assisted activation step on surface area and CO₂ uptake. The study also evaluates the effect of various chemical agents used during activation. Date palm leaves, when carbonized under moderate operational conditions, perform better than other biomass precursors.

In contrast, while Elega biomass requires a lower carbonization temperature, it still demands extensive pretreatment and does not yield optimal results. Therefore, date palm leaves are an excellent candidate for activated carbon (AC) production. They require minimal pretreatment and moderate conditions, and, importantly, are abundant in large quantities, especially in the Middle East, making them a sustainable and cost-effective source for AC preparation.

**Table 9:** Comparative analysis between the current study and other carbon sources, detailing their optimal carbonization conditions and the influence of the microwave activation step on activated carbon (AC) properties and CO_2_ adsorption capacity.

Carbon source	Pyrolysis Condition	Activation Condition	Adsorption Condition	CO_2_ Removal	Surface area m^2^/g	Refs.
				mg/gm		
Microalgae powders (chlorella and spirulina)	• 800 °C	• 2:1 KOH	25 ^○^C ,1 bar	184.8	602	^35^
	• 20 min.	• 700 W				
	• 1:1 Urea	• 15 min		157.1	510	
	• 350 °C					
	• 90 min					
Pecan nutshell	• 10 ^○^C/min	• 1:1 KOH	25^○^C, 1 bar	146	887	^15^
	• 600 ^○^C	• 300 W				
	• 60 min	• 8 min				
Date palm leaves	• 450 ^○^C	• 1.5:1 K_2_CO_3_	25 ^○^C, 1 bar	126.7	411	Current work
	• 10 ^○^C/min	• 850 W				
	• 90 min	• 500 ^○^C				
		• 7 min				
Palm kernel shells	• 700 °C	• 1:1 K_2_CO_3_	30 ^○^C, 1 bar	122	322.5	^36^
	• 10 °C/min	• 11 min				
	• 120 min	• 400 W				
		• CO_2_				
Micro-crystalline cellulose	• 500 ◦C	• 4:1commercil AC (Norit R),	25 ^○^C, 1 bar	76.13	510	^14^
	• 10 ^○^C/min	• 400 ^○^C				
	• 60 min	• 60 min				
Bamboo	• 600 °C	• 1:20 lignin	25^○^C, 1 bar	60	377.32	^37^
	• 300 min	• 600 W				
		• 20 min				

### A comparative analysis of energy and time efficiency between two activation techniques

A key factor in evaluating the efficiency and economic feasibility of a process is energy consumption. Given the current acceleration of the energy crisis, it is essential to investigate the differences in the amount of energy required for each technique.

**Fig. 9:**
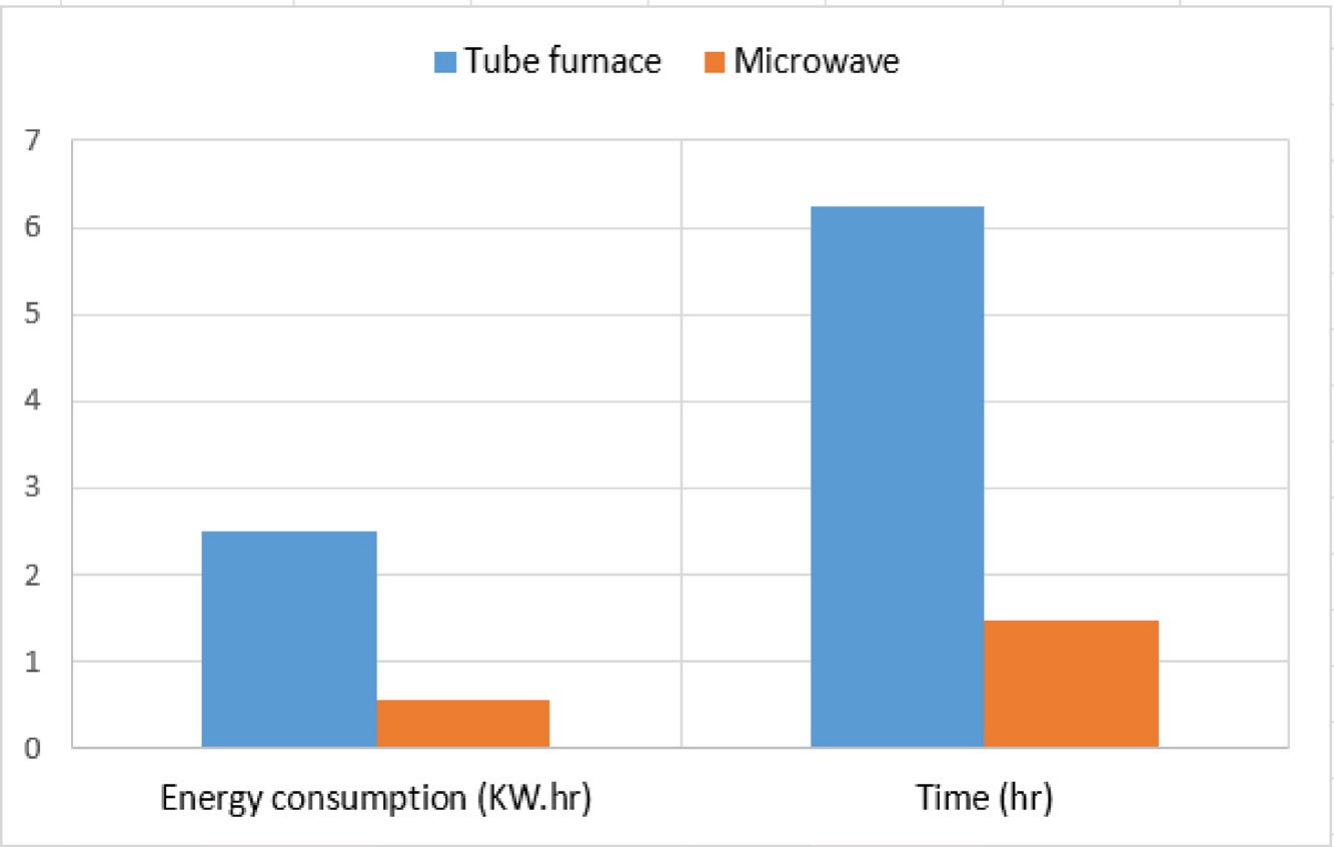
Comparative analysis of energy and time efficiency between tube furnace & microwave.

Based on the analysis of the collected data in the present study, the employed tube furnace operated with an input power of 2000 watts. It required approximately 15 min, with a heating rate of 10 °C/min, to transition the temperature from 450 °C (carbonization step) to 600 °C (activation temperature). The furnace then maintained a stable temperature of activation for 60 min. In contrast, during microwave activation under optimal conditions, the furnace operated at 850 watts for 32 min to reach the desired temperature of 500 °C. after which it maintained this temperature for only 7 min. As illustrated in Figure. 9, in terms of energy consumption, the microwave technique required approximately 4.5 times less energy than the conventional method. Considering the cooling times of 6 h for the tube furnace and 50 min for the microwave, the overall activation and cooling time under an inert gas atmosphere using the microwave was less than a quarter of the time required by the conventional technique. Correspondingly, utilizing microwave power during the activation step is not only crucial for enhancing product quality but also plays a significant role in reducing overall energy consumption and shortening the processing time^38^.

## Conclusion

We demonstrated that mesoporous activated carbons derived from date palm leaves, produced by integrating conventional carbonization with microwave activation in a hybrid system, significantly improve synthesis conditions, AC characteristics, and CO₂ adsorption capacity. The effectiveness of this hybrid method was evaluated using Response Surface Methodology and the Box-Behnken model, which identified microwave power and activation temperature as the most significant factors on AC quality. The optimal conditions of 850 W microwave power, 600 °C activation temperature, and a 7-minute activation time resulted in a notable improvement in AC textural properties, achieving a CO₂ adsorption capacity of 126.7 mg/g while significantly reducing energy and time demands to about 25%. The resulting AC offers an eco-friendly solution for carbon capture and environmental remediation, supporting broader efforts in environmental protection and resource optimization. These findings highlight the potential of combining hybrid carbonization and microwave activation for enhanced CO₂ adsorption efficiency, with future studies focusing on optimizing pressure swing adsorption units to further improve scalability.

## Supplementary Information

Below is the link to the electronic supplementary material.

Supplementary Material 1

Supplementary Material 2

## Data Availability

The datasets used or analysed during the current study are available from the corresponding author upon reasonable request.

## References

[1] Mahrous, A. A. et al. *Effect of Date Palm (Phoenix Dactylifera L.) Leaves on Productive Performance of Growing Lambs*53pp.1–8 (Tropical Animal Health and Production, 2021). 10.1007/s11250-020-02493-2 PMC7785545 33400015

[2] Zafar, S. Biomass Potential of Date Palm Wastes. EcoMENA. (2022). Retrieved from https://www.ecomena.org

[3] Faiad, A. et al. Date palm tree waste recycling: treatment and processing for potential engineering applications. Sustainability, 14(3), p.1134. (2022).

[4] Hagemann, N. et al. Activated carbon, biochar and charcoal: linkages and synergies across pyrogenic carbon’s ABC s. Water, 10(2), p.182. (2018).

[5] Al-Janabi, S. K., Barron, A. R., Shabbani, H. J. K., Othman, M. R. & Kim, J. Advances in hydrogen production from sustainable recourses through biological and thermochemical pathways: review and bibliometric analysis. *Int. J. Hydrog. Energy*. **60**, 28–45 (2024).

[6] Aloud, S. S. et al. Adsorption of Pb2 + by activated carbon produced by microwave-assisted K2CO3 activation of date palm leaf sheath fibres. Water, 15(22), p.3905. (2023).

[7] Sathish, S., Nirmala, R., Kim, H. Y. & Navamathavan, R. Deriving activated carbon using microwave combustion technique and its energy storage applications: a topical review. *Carbon Lett.* **32** (5), 1151–1171 (2022).

[8] Hu, Q. et al. Microwave technology: A novel approach to the transformation of natural metabolites. *Chin. Med.* **16**, 1–22 (2021). 34530887 10.1186/s13020-021-00500-8 PMC8444431

[9] Grekov, D., Pré, P. & Alappat, B. J. Microwave mode of heating in the preparation of porous carbon materials for adsorption and energy storage applications–an overview. Renewable and Sustainable Energy Reviews, 124, p.109743. (2020).

[10] Kumar, R., Sahoo, S., Joanni, E. & Singh, R. K. A review on the current research on microwave processing techniques applied to graphene-based supercapacitor electrodes: an emerging approach beyond conventional heating. *J. Energy Chem.* **74**, 252–282 (2022).

[11] Lam, S. S. & Chase, H. A. A review on waste to energy processes using microwave pyrolysis. *Energies* **5** (10), 4209–4232 (2012).

[12] Sasi Kumar, N., Denys Grekov, P., Pré, B. J. & Alappat Microwave mode of heating in the preparation of porous carbon materials for adsorption and energy storage applications – An overview, Renewable and Sustainable Energy Reviews, Volume 124, 109743, ISSN 1364 0321. (2020).

[13] Durán-Jiménez, G. et al. Rapid, simple and sustainable synthesis of ultra-microporous carbons with high performance for CO2 uptake, via microwave heating. Chemical Engineering Journal, 388, p.124309. (2020).

[14] Biti, S., McCue, A. J., Dionisi, D., Graça, I. & Martín, C. F. Greener carbon capture using microwave heating for the development of cellulose-based adsorbents. Fuel, 358, p.130246. (2024).

[15] Durán-Jiménez, G. et al. Simultaneous conventional and microwave heating for the synthesis of adsorbents for CO2 capture: Comparative study to pristine technologies. Chemical Engineering Journal, 438, p.135549. (2022).

[16] Shi, C. et al. Efficient heating of activated carbon in microwave field. C, 9(2), p.48. (2023).

[17] Ao, W. et al. Microwave assisted Preparation of activated carbon from biomass: A review. *Renew. Sustain. Energy Rev.* **92**, 958–979 (2018).

[18] Foo, K. Y. & Hameed, B. H. Preparation, characterization and evaluation of adsorptive properties of orange Peel based activated carbon via microwave induced K2CO3 activation. *Bioresour. Technol.* **104**, 679–686 (2012). 22101073 10.1016/j.biortech.2011.10.005

[19] Jasri, K. et al. Mesoporous activated carbon produced from mixed wastes of oil palm frond and palm kernel shell using microwave radiation-assisted K2CO3 activation for methylene blue dye removal: Optimization by response surface methodology. Diamond and Related Materials, 131, p.109581. (2023).

[20] Zhu, L., Zhao, N., Tong, L. & Lv, Y. Structural and adsorption characteristics of potassium carbonate activated Biochar. *RSC Adv.* **8** (37), 21012–21019 (2018). 35542323 10.1039/c8ra03335h PMC9080865

[21] Wu, W. et al. Synthesis and characterization of magnetic K2CO3-activated carbon produced from bamboo shoot for the adsorption of Rhodamine b and CO2 capture. Fuel, 332, p.126107. (2023).

[22] Illán-Gómez, M. J., Garcia-Garcia, A., Salinas-Martinez de Lecea, C. & Linares-Solano, A. Activated carbons from Spanish coals. 2. Chemical activation. *Energy Fuels*. **10** (5), 1108–1114 (1996).

[23] Salema, A. A. Farid Nasir ani, microwave induced pyrolysis of oil palm biomass. *Bioresour. Technol.* **102** (Issue 3), 3388–3395 (2011). 20970995 10.1016/j.biortech.2010.09.115

[24] Sevilla, M., Díez, N. & Fuertes, A. B. More sustainable chemical activation strategies for the production of porous carbons. *ChemSusChem* **14** (1), 94–117 (2021). 33047490 10.1002/cssc.202001838

[25] Bläker, C., Muthmann, J., Pasel, C. & Bathen, D. Characterization of activated carbon adsorbents–state of the Art and novel approaches. *ChemBioEng Reviews*. **6** (4), 119–138 (2019).

[26] Cheng, S. Zeng, X. & Liu, P. One-step synthesis of magnetic N-doped carbon nanotubes derived from waste plastics for effective Cr(VI) removal, Arabian journal of chemistry, 17, issue 10,2024,105956, ISSN 1878–5352. (2024)

[27] Melliti, A., Srivastava, V., Kheriji, J., Sillanpää, M. & Hamrouni, B. Date Palm Fiber as a novel precursor for porous activated carbon: Optimization, characterization and its application as Tylosin antibiotic scavenger from aqueous solution. Surfaces and Interfaces, 24, p.101047. (2021).

[28] Ren, X. et al. Challenges and opportunities in microwave-assisted catalytic pyrolysis of biomass: A review. Applied Energy, 315, p.118970. (2022).

[29] Al-Ghouti, M. A. & Da’ana, D. A. Guidelines for the use and interpretation of adsorption isotherm models: A review. *J. Hazard. Mater.* **393**, 122383 (2020). 32369889 10.1016/j.jhazmat.2020.122383

[30] Zhang, S. et al. *The Role and Mechanism of K2CO3 and Fe3O4 in the Preparation of Magnetic Peanut Shell Based Activated Carbon*295pp.152–160 (Powder Technology, 2016).

[31] Guo, Y. et al. Porous activated carbons derived from waste sugarcane Bagasse for CO2 adsorption. *Chem. Eng. J.* **381**, 122736 (2020).

[32] Villota, S. M. et al. Microwave-assisted activation of waste cocoa pod husk by H3PO4 and KOH—comparative insight into textural properties and pore development. *ACS Omega*. **4** (4), 7088–7095 (2019).

[33] Zaini, M. A. A. & Kamaruddin, M. J. Critical issues in microwave-assisted activated carbon Preparation. *J. Anal. Appl. Pyrol.* **101**, 238–241 (2013).

[34] Baytar, O., Şahin, Ö. & Saka, C. Sequential application of microwave and conventional heating methods for Preparation of activated carbon from biomass and its methylene blue adsorption. *Appl. Therm. Eng.* **138**, 542–551 (2018).

[35] Shi, S. & Liu, Y. Nitrogen-doped activated carbons derived from microalgae pyrolysis by-products by microwave/koh activation for CO2 adsorption. *Fuel* **306**, 121762 (2021).

[36] Hamza, U. D., Nasri, N. S., Amin, N. A. S., Mohammed, J. & Zain, H. M. Microwave assisted K2CO3 palm shell activated carbon as sorbent for CO2 adsorption application. *Jurnal Teknologi*, (Sciences and Engineering). 78. 8 – 3. (2016)

[37] Zhang, X. et al. Lignin-impregnated biochar assisted with microwave irradiation for CO2 capture: adsorption performance and mechanism. Biochar, 6(1), p.22. (2024).

[38] Li, J. et al. Rapid preparation strategy of highly microporous activated carbons for gas adsorption, via tunable-energy-density microwave heating. Renewable Energy, 225, p.120260. (2024).

