# Supplementary material for: Microwave-assisted enhanced activation of date palm leaf char for optimized CO2 adsorption
Source: Sci Rep. 2025 Oct 7;15:34928. doi: 10.1038/s41598-025-18683-7 (PMC12504704; doi:10.1038/s41598-025-18683-7)
Supplement: Supplementary file 1 — Supplementary Material 1 [file 41598_2025_18683_MOESM1_ESM.docx]

| Run Order | Actual Value | | Predicted Value | Residual | | Leverage | | | Internally Studentized Residuals | Externally Studentized Residuals | | Cook's Distance | Influence on Fitted Value DFFITS | | Standard Order | | |
| --- | --- | --- | --- | --- | --- | --- | --- | --- | --- | --- | --- | --- | --- | --- | --- | --- | --- |
| 1 | 12 123 | 118.53 | | | 4.47 | | 0.450 | 1.127 | | | 1.149 | 0.173 | | 1.039 | | 9 |  |
| 2 | 107.00 | | 101.53 | 5.47 | | 0.450 | | | 1.378 | 1.477 | | 0.259 | 1.336 | | 4 | | |
| 3 | 103.00 | | 109.28 | -6.28 | | 0.450 | | | -1.580 | -1.782 | | 0.340 | -1.612 | | 11 | | |
| 4 | 129.00 | | 126.70 | 2.30 | | 0.300 | | | 0.513 | 0.488 | | 0.019 | 0.320 | | 14 | | |
| 5 | 98.00 | | 102.03 | -4.03 | | 0.450 | | | -1.013 | -1.015 | | 0.140 | -0.918 | | 2 | | |
| 6 | 108.00 | | 108.78 | -0.7750 | | 0.450 | | | -0.195 | -0.183 | | 0.005 | -0.165 | | 12 | | |
| 7 | 130.00 | | 126.70 | 3.30 | | 0.300 | | | 0.736 | 0.714 | | 0.039 | 0.467 | | 13 | | |
| 8 | 130.00 | | 129.78 | 0.2250 | | 0.450 | | | 0.057 | 0.053 | | 0.000 | 0.048 | | 1 | | |
| 9 | 86.00 | | 84.10 | 1.90 | | 0.450 | | | 0.478 | 0.454 | | 0.031 | 0.411 | | 8 | | |
| 10 | 122.00 | | 129.28 | -7.28 | | 0.450 | | | -1.832 | -2.249 | | 0.458 | -2.034 | | 3 | | |
| 11 | 115.00 | | 118.03 | -3.03 | | 0.450 | | | -0.762 | -0.740 | | 0.079 | -0.669 | | 10 | | |
| 12 | 123.00 | | 121.10 | 1.90 | | 0.450 | | | 0.478 | 0.454 | | 0.031 | 0.411 | | 5 | | |
| 13 | 117.00 | | 111.85 | 5.15 | | 0.450 | | | 1.297 | 1.365 | | 0.229 | 1.234 | | 7 | | |

Current Transform: CO_2_ Uptake

Recommended Transform: None

Best Lambda: 0.86

95% CI Low: –2.41

95% CI High: 5.04

| Run Order | Actual Value | Predicted Value | Residual | Leverage | Internally Studentized Residuals | Externally Studentized Residuals | Cook's Distance | Influence on Fitted Value DFFITS | Standard Order |
| --- | --- | --- | --- | --- | --- | --- | --- | --- | --- |
| 1 | 16.00 | 16.71 | -0.7125 | 0.321 | -0.813 | -0.794 | 0.052 | -0.546 | 9 |
| 2 | 18.00 | 18.63 | -0.6250 | 0.571 | -0.897 | -0.885 | 0.179 | -1.022 | 4 |
| 3 | 19.00 | 18.71 | 0.2875 | 0.321 | 0.328 | 0.309 | 0.008 | 0.213 | 11 |
| 4 | 20.00 | 18.05 | 1.95 | 0.071 | 1.902 | 2.403 | 0.046 | 0.666 | 14 |
| 5 | 20.00 | 20.80 | -0.8000 | 0.571 | -1.148 | -1.175 | 0.293 | -1.357 | 2 |
| 6 | 19.00 | 19.39 | -0.3875 | 0.321 | -0.442 | -0.419 | 0.015 | -0.288 | 12 |
| 7 | 17.00 | 18.05 | -1.05 | 0.071 | -1.024 | -1.027 | 0.013 | -0.285 | 13 |
| 8 | 15.00 | 14.63 | 0.3750 | 0.571 | 0.538 | 0.513 | 0.064 | 0.592 | 1 |
| 9 | 20.00 | 19.46 | 0.5375 | 0.571 | 0.772 | 0.750 | 0.132 | 0.866 | 8 |
| 10 | 18.70 | 18.15 | 0.5500 | 0.571 | 0.789 | 0.769 | 0.139 | 0.888 | 3 |
| 11 | 17.00 | 17.39 | -0.3875 | 0.321 | -0.442 | -0.419 | 0.015 | -0.288 | 10 |
| 12 | 14.00 | 14.14 | -0.1375 | 0.571 | -0.197 | -0.185 | 0.009 | -0.214 | 5 |
| 13 | 18.00 | 18.64 | -0.6375 | 0.571 | -0.915 | -0.905 | 0.186 | -1.045 | 7 |
| 14 | 21.00 | 19.96 | 1.04 | 0.571 | 1.489 | 1.639 | 0.493 | 1.892 | 6 |

Current Transform: yield

Recommended Transform: None

Best Lambda: –1.03

95% CI Low: –5.23

95% CI High: 3.16

| Run Order | Actual Value | Predicted Value | Residual | Leverage | | Internally Studentized Residuals | | Externally Studentized Residuals | | Cook's Distance | | Influence on Fitted Value DFFITS | | Standard Order | |  |
| --- | --- | --- | --- | --- | --- | --- | --- | --- | --- | --- | --- | --- | --- | --- | --- | --- |
| 1 | 28.00 | 28.05 | -0.0536 | | 0.321 | | -0.041 | | -0.039 | | 0.000 | | -0.027 | | 9 | |
| 2 | 23.00 | 23.18 | -0.1786 | | 0.321 | | -0.137 | | -0.130 | | 0.002 | | -0.089 | | 4 | |
| 3 | 37.00 | 38.05 | -1.05 | | 0.321 | | -0.806 | | -0.791 | | 0.077 | | -0.544 | | 11 | |
| 4 | 32.00 | 32.43 | -0.4286 | | 0.071 | | -0.280 | | -0.267 | | 0.002 | | -0.074 | | 14 | |
| 5 | 24.00 | 24.43 | -0.4286 | | 0.321 | | -0.328 | | -0.313 | | 0.013 | | -0.215 | | 2 | |
| 6 | 38.00 | 36.80 | 1.20 | | 0.321 | | 0.915 | | 0.907 | | 0.099 | | 0.624 | | 12 | |
| 7 | 35.00 | 32.43 | 2.57 | | 0.071 | | 1.682 | | 1.884 | | 0.054 | | 0.522 | | 13 | |
| 8 | 42.00 | 41.68 | 0.3214 | | 0.321 | | 0.246 | | 0.234 | | 0.007 | | 0.161 | | 1 | |
| 9 | 27.00 | 28.80 | -1.80 | | 0.321 | | -1.380 | | -1.455 | | 0.225 | | -1.001 | | 8 | |
| 10 | 38.00 | 40.43 | -2.43 | | 0.321 | | -1.858 | | -2.178 | | 0.409 | | -1.499 | | 3 | |
| 11 | 27.00 | 26.80 | 0.1964 | | 0.321 | | 0.150 | | 0.143 | | 0.003 | | 0.098 | | 10 | |
| 12 | 35.00 | 36.05 | -1.05 | | 0.321 | | -0.806 | | -0.791 | | 0.077 | | -0.544 | | 5 | |
| 13 | 48.00 | 46.05 | 1.95 | | 0.321 | | 1.489 | | 1.601 | | 0.263 | | 1.102 | | 7 | |
| 14 | 20.00 | 18.80 | 1.20 | | 0.321 | | 0.915 | | 0.907 | | 0.099 | | 0.624 | | 6 | |

Current Transform: Ramp time

Recommended Transform: None

Best Lambda: 0.39

95% CI Low: –0.44

95% CI High: 1.22
