# Supplementary material for: Microwave-assisted enhanced activation of date palm leaf char for optimized CO2 adsorption
Source: Sci Rep. 2025 Oct 7;15:34928. doi: 10.1038/s41598-025-18683-7 (PMC12504704; doi:10.1038/s41598-025-18683-7)
Supplement: Supplementary file 2 — Supplementary Material 2 [file 41598_2025_18683_MOESM2_ESM.docx]

**Highlights**

- Activated carbon was synthesized from date palm leaves (DPL).
- TGA was used to evaluate thermal stability and CO₂ uptake.
- Optimal CO₂ adsorption conditions were systematically investigated.
- Activated carbon was comprehensively characterized.
- Microwave and tube furnace methods were compared for energy efficiency.
